# Metagenomic and microbiological analyses of historical manuscripts for bacterial community profiling and bacteria-related biodeterioration assessment

**DOI:** 10.15698/mic2026.03.871

**Published:** 2026-03-01

**Authors:** Esra Keles, Ozge Celik

**Affiliations:** 1 Department of Conservation and Archive, The Manuscript Institution of Türkiye, Istanbul, Turkiye; 2 Department of Molecular Biology and Genetics, Faculty of Science and Letter, T.C. Istanbul Kültür University, Istanbul, Turkiye

**Keywords:** Historical manuscripts, biodeterioration, metagenomics, Illumina sequencing, bacterial community profiling

## Abstract

Bacteria are important agents in the biodeterioration of cultural heritage objects, including historical manuscripts. Characterizing bacterial communities and generating robust microbiological data has therefore become crucial for conservation and restoration strategies. In this study, we investigated the bacterial communities associated with biodeterioration in six historical manuscripts using both culture-dependent and culture-independent (Illumina MiSeq) approaches. Culture-dependent methods yielded only 16 viable and culturable isolates, highlighting the limitations of traditional techniques. In contrast, metagenomic analysis revealed a far richer and more diverse bacterial community, capturing both living and non-living microbial traces accumulated over centuries. Bacterial genera with known cellulolytic and/or proteolytic activities, such as *Bacillus, Stenotrophomonas, Pseudomonas* and *Acinetobacter*, were identified as part of a core microbiome commonly associated with paper deterioration. High abundances of gut-associated bacteria (*Prevotella, Faecalibacterium, Bacteroides, Porphyromonas*) and human-related taxa (*Staphylococcus, Streptococcus, Cutibacterium*) indicated extensive historical human handling. A notable finding was the detection of *Pseudonocardia broussonetiae*, an endophytic bacterium associated with paper mulberry (*Broussonetia papyrifera*), suggesting the possible use of this plant as a papermaking material in one manuscript. This represents an important contribution to understanding Islamic paper production. Overall, our results demonstrate that effective conservation strategies require a detailed understanding of each manuscript’s microbial ecology, together with evidence of past environmental conditions, handling history, and production materials.

## INTRODUCTION

Manuscripts are historical books and documents created by hand, without the use of printing technology. As an important part of Türkiye’s cultural heritage, manuscripts provide valuable insights into the periods and regions in which they were produced. However, many of these historically and culturally significant manuscripts have undergone biodeterioration caused by biological agents such as microorganisms, insects, and rodents, leading to both aesthetic and structural damage.

Microorganisms colonize the organic and inorganic components of manuscript through diverse enzymatic activities including proteolytic enzymes and cellulolytic enzymes—endogluconases, exogluconases, and β -glycosidases—particularly under favorable environmental conditions 1, 2 and owing to their ability to grow at low water activity (aw) 3, 4. Cellulose, the main component of paper, and collagen, the structural protein of parchment, are particularly susceptible to microbial degradation 5, 6. Additionally, adhesives, fillers, and trace elements (K, Mg, S, P) originating from manufacturing materials or environmental sources can affect the susceptibility of manuscripts to microbial attack. The presence of water is also an important factor in promoting the growth of microorganisms on paper 2. Over time, microbial metabolism can lower paper pH, accelerate acid hydrolysis, and cause discoloration, staining, foxing, fiber disruption, and loss of mechanical strength 7–10.

In general, the contamination of library and archival materials has long been assumed to be primarily caused by the presence of fungi. Although fungi are important producers of cellulolytic enzymes, bacteria possess a greater potential for cellulase synthesis due to their higher growth rates compared to fungi 11. Recent studies demonstrate that bacteria also play a significant role in the deterioration of paper, leather, inks, and adhesives 12–16. Fungal colonization and cellulose biodegradation may also create secondary conditions that facilitate subsequent bacterial growth on altered substrates 17. Consequently, bacterial communities can be equally destructive under specific microenvironmental conditions, such as fluctuating humidity, intensive handling, and salt- or starch-rich substrates.

Bacterial contamination of historical books arises from multiple sources, including airborne dust, human skin contact, and vectors such as insects and mites 18–21. Krakova *et al.* 18 demonstrated that microbial communities isolated from library materials are closely linked to the environmental conditions of libraries. These conditions include the microbial load of indoor air, ventilation systems, dust accumulation, and temperature and relative humidity. Contamination predominantly occurs through airborne pathways, often facilitated by damp and leaking walls, the presence of mouldy food, and inadequate ventilation systems in libraries 19, 20. Among the bacteria detected on paper, *Bacillus spp*. are frequently isolated and have been associated with paper deterioration, although in some cases they may simply reflect dust deposition rather than active degradation 21. Human-associated genera such as *Staphylococcus* and *Streptococcus* are also commonly recovered from manuscript surfaces, consistent with their presence in the skin, saliva, and ocular microbiomes 22–24. Insects and mites play an important role in the dissemination of microorganisms and their spores. Pavlovic *et al.* 15 reported that the bacterial communities identified in their study showed similarities to the microbiomes of certain species of Lepidoptera and Coleoptera 25, which are known to be among the most harmful insect pests in museums and libraries. Bacterial genera such as *Staphylococcus*, *Bacillus*, *Micrococcus*, and *Pseudomonas* identified in their study are commonly associated with these insect orders.

Comprehensive identification of microbial communities colonizing manuscripts and distinguishing between passive deposition and active biodegradation is therefore essential for interpreting microbial community data in a conservation context. Approaches have been employed to characterize these communities, generally falling into two major categories: culture-dependent and culture-independent methods. Microorganisms isolated through culture-dependent approaches have typically been identified using molecular techniques based on the amplification and sequencing of bacterial 16S rRNA genes 10, 18, 26, 27. Culture-independent approaches such as denaturing gradient gel electrophoresis (DGGE), clone libraries, and more recently next-generation sequencing (NGS) have expanded our view of these communities 32–35. However, culture-based techniques and DGGE/clone library analyses are insufficient to capture the full diversity and relative abundances of microbial taxa inhabiting manuscripts, particularly viable-but-non-culturable (VBNC) populations 30, 31.

In recent years, Illumina-based 16S rRNA gene amplicon sequencing has enabled high-resolution, culture-independent profiling of bacterial communities in library materials and historical books 22, 32–34. These approaches provide information not only on the presence but also on the relative abundance, diversity, and potential ecological roles of bacterial taxa, and allow temporal shifts in microbial communities to be monitored. Despite this progress, the bacterial microbiomes of historical manuscripts produced within the Islamic cultural and scribal tradition remain largely unexplored. Distinct material characteristics—such as gelatin- and starch-based sizing, gum Arabic inks, natural dyes, alum-tawed leather bindings, and region-specific papermaking techniques—create a unique biochemical landscape that may select for specific bacterial assemblages, yet this relationship is still poorly understood. To our knowledge, no previous study has combined metagenomic sequencing with culture-based microbiology to characterize the bacterial diversity of Islamic manuscripts spanning the 13th–18th centuries.

The present study addresses this knowledge gap by combining Illumina MiSeq-based 16S rRNA gene amplicon sequencing with targeted microbiological isolation to characterize bacterial communities inhabiting six biodeteriorated Islamic manuscripts from the Süleymaniye Manuscripts Library (Istanbul, Türkiye). Rather than directly quantifying functional biodegradation through enzymatic assays, our primary objective was to map the taxonomic structure and ecological signals of bacterial colonization and to identify bacterial groups previously associated with deterioration phenomena in the literature. By documenting both culturable and non-culturable taxa, this work provides a foundational dataset for understanding bacterial contributions to manuscript stability and offers a methodological framework for future research on biodeterioration dynamics in Islamic and global documentary heritage.

## RESULTS

### Culture-dependent analysis

A total of 16 bacteria were isolated from six manuscripts using culture-dependent methods on TSA media. These bacteria were characterized and identified at the species level by 16S rRNA gene amplification and sequencing analysis. Their identification frequencies ranged from 90.72% to 100% when compared with reference genes in the NCBI database. Isolates I1, I3, I5 and I8 showed similarity (98.55–99.13%) to *Niallia circulans*, Isolates I6 and I7 showed similarity (99.87) to *Micrococcus luteus*, while I4, I15 and I14 showed similarity (99.85–100) to *Priestia megaterium*. The remaining isolates shared identity (90.72–100) with *Ornithinibacillus scarpharcae, Ferdinandcohnia humi, Neobacillus niacini, Psychrobacter pulmonis, Microbacterium foliorum* and *Bacillus haynesii.* (The results are provided in Table 1.)

**Table 1:** Identification of culturable bacterial isolates recovered from historic manuscripts based on 16S rRNA gene sequence analysis .

**Manuscripts Code**	**Isolate Number**	**Closest relative species**	**Similarity (%)**	**NCBI Accession Numbers of reference genes**
**T1**	I5	*Niallia circulans*	99.11	NR_112632.1
	I6	*Micrococcus luteus*	99.87	NR_075062.2
	I7	*Micrococcus luteus*	99.87	NR_075062.2

**T2**	I1	*Niallia circulans*	98.55	NR_112632.1
	I2	*Fredinandcohnia humi*	98.46	NR_025626.1
	I3	*Niallia circulans*	99.13	NR_112632.1
	I4	*Priestia megaterium*	99.85	NR_117473.1

**T3**	I8	*Niallia cİrculans*	98.86	NR_112632.1
	I14	*Priestia megaterium*	99.86	NR_117473.1

**T4**	I11	*Neobacillus niacini*	98.85	NR_113777.1
	I12	*Ornithinibacillus scarpharcae*	90.72	NR_117927.1
	I13	*Psychrobacter pulmonis*	100	NR_118026.1

**T5**	I10	*Bacillus haynesii*	99.71	NR_157609.1
	I15	*Priestia megaterium*	100	NR_117473.1
	I16	*Ornithinibacillus scarpharcae*	100	NR_117927.1

**T6**	I9	*Microbacterium foliorum*	99.24	NR_025368.1

The closest relative species, percentage sequence similarity, and corresponding NCBI accession numbers are shown for each isolate. Manuscript codes indicate the source manuscript, and isolate numbers refer to individual bacterial strains recovered through culture-dependent methods.

### Culture-independent analysis (metagenomics)

DNA extraction performed on paper fragments obtained from the six manuscripts yielded sufficient and comparable amounts of nucleic acid, with concentrations ranging from 25.2 to 33.9 μ g/ μ L. The extracted DNA was of adequate quality for downstream analyses, enabling successful amplification and sequencing. Illumina MiSeq high-throughput sequencing generated a substantial number of reads per sample, though with considerable variation across the dataset. Sample T2 produced the highest number of reads (378,425), whereas T5 showed the lowest sequencing depth (46,232 reads). The Phred quality filtering confirmed the overall reliability of the dataset: Q20 values ranged between 99.38% and 100%, while Q30 values spanned from 90% to 96.15%, with T3 showing the highest read quality. Taken together, these metrics indicate that the sequencing effort was successful and provided high-fidelity reads suitable for robust taxonomic analyses. Based on the sequencing data, taxonomic assignments were performed and operational taxonomic units (OTUs) were generated, enabling the construction of filum-level and genus-level taxonomic profile. Only OTUs with exact (100%) sequence matches were assigned at the species level, and all remaining taxa are presented at the genus level. (Table 2)

**Figure 1:**
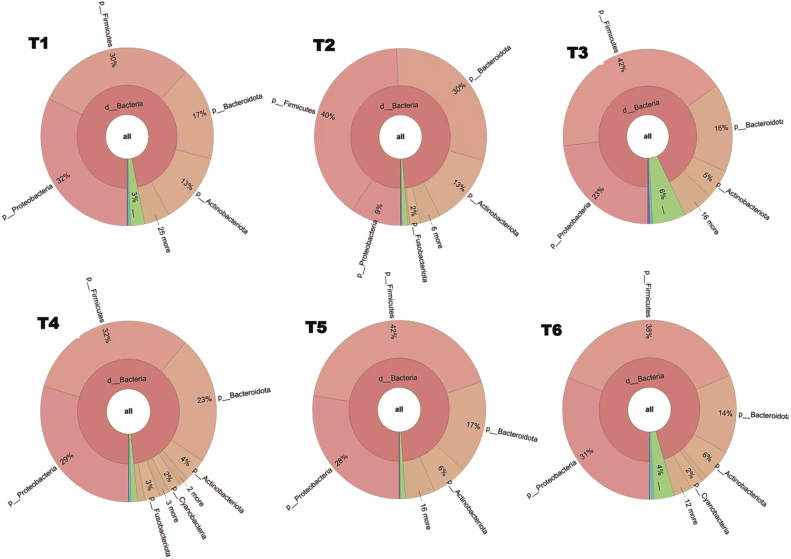
Krona charts illustrating the phylum-level Taxonomic composition of bacterial communities associated with historic manuscript samples based on Illumina MiSeq sequencing. Circular hierarchical plots illustrating the relative abundance of bacterial taxa detected in six historic manuscript samples (T1–T6) based on 16S rRNA gene amplicon sequencing. The plots display taxonomic assignments from the domain level (*Bacteria*) to lower taxonomic ranks, highlighting the proportional distribution of dominant bacterial phyla, including *Proteobacteria*, *Firmicutes*, *Bacteroidota*, and *Actinobacteriota*. Relative abundances are represented as percentages, and taxa accounting for low proportions of the community are grouped accordingly. The visualizations were generated from normalized OTU tables using Krona to facilitate comparative analysis of microbial community structure across samples.

At the phylum level, all manuscript samples (T1–T6) were dominated by Firmicutes, Proteobacteria, Bacteroidota, and Actinobacteria (Figure 1), with Firmicutes representing the most abundant group (30–42%). Proteobacteria (9–32%) and Bacteroidota (14–30%) showed marked variability among samples, whereas Actinobacteria remained consistently lower (4–13%). Fusobacteriota occurred only at minor proportions (<3%). Despite sharing a common phylum-level composition, the manuscripts exhibited distinct relative abundance patterns.

**Table 2:** Summary of Illumina MiSeq sequencing output, quality filtering, and OTU classification at the genus level .

**Sample**	**Input**	**Filtered**	**Merged**	**Q20 (%)**	**Q30 (%)**	**OTU’s in genus level**
**T1**	205584	182932	145233	99.9	95.6	495
**T2**	378425	282551	193395	99.38	90	540
**T3**	58498	46341	34553	100	96.15	280
**T4**	60537	47527	36523	99.9	95.16	337
**T5**	46232	35911	26520	99.9	95.14	226
**T6**	95562	83730	79918	100	97.65	338

Sequencing statistics for six manuscript samples (T1–T6) generated by Illumina MiSeq analysis are shown, including the number of raw input reads, reads retained after quality filtering, and merged paired-end reads. Quality metrics are expressed as the percentage of bases with Phred quality scores ≥ 20 (Q20) and ≥ 30 (Q30). The number of Operational Taxonomic Units (OTUs) classified at the genus level is reported for each sample following downstream bioinformatic processing.

**Figure 2:**
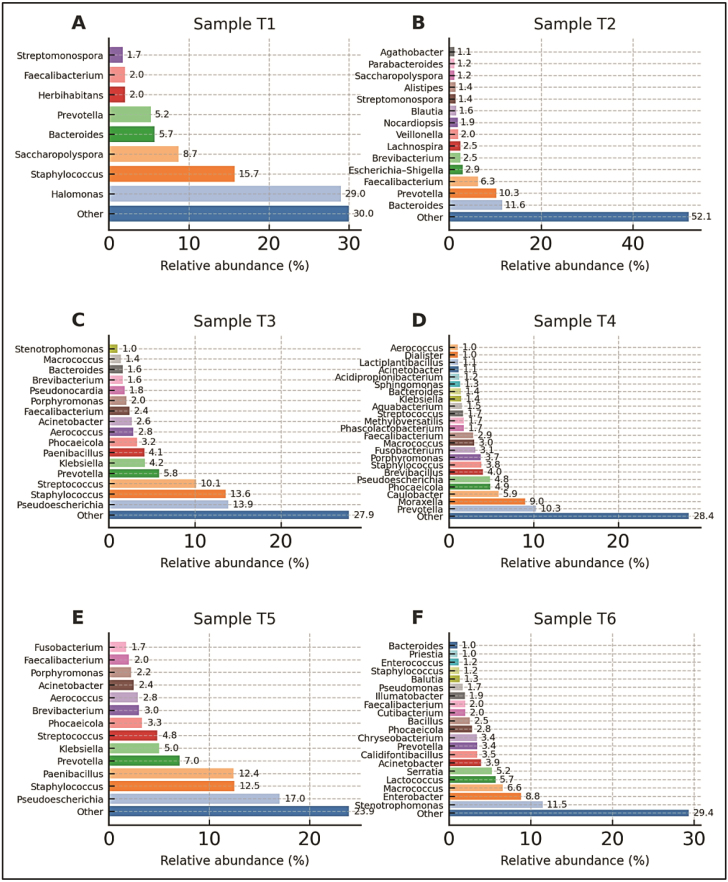
Genus-level bacterial community composition of historic manuscript samples based on Illumina MiSeq sequencing. Horizontal bar charts showing the relative abundance (%) of dominant bacterial genera identified in six historic manuscript samples: **(A)** Sample T1, **(B)** Sample T2, **(C)** Sample T3, **(D)** Sample T4, **(E)** Sample T5, and **(F)** Sample T6. Taxonomic profiles were generated from normalized 16S rRNA gene amplicon sequencing data obtained using the Illumina MiSeq platform. Only genera exceeding 1% relative abundance are shown individually, while taxa with lower abundances are grouped under “Other”.

At the genus level, distinct bacterial community structures were observed across the six manuscript samples (T1–T6) (Figure 1), reflecting differences in taxonomic composition as well as sequence similarity and phylogenetic homology among detected taxa. A total of 495 OTUs were identified in T1, 540 in T2, 280 in T3, 337 in T4, 226 in T5, and 338 in T6. Reads that could not be assigned to OTUs constituted a small fraction of each dataset, ranging from 210 (T5) to 2,898 (T1). OTUs with a relative abundance below 1% were pooled into an “other” category due to their low representation. Based on >1% relative abundance thresholds, eight dominant genera were identified in T1, 14 in T2, 16 in T3, 23 in T4, 13 in T5, and 20 in T6. (Figure 2)

In sample T1, the dominant phyla were Proteobacteria (32%), Firmicutes (30%), Bacteroidota (17%), and Actinobacteria (13%). Within Proteobacteria, Gammaproteobacteria were primarily represented by *Halomonas*, accounting for nearly 29% of total reads. Actinobacterial taxa included *Saccharopolyspora*, *Herbihabitans*, and *Streptomonospora*, which share phylogenetic homology. Firmicutes were dominated by *Staphylococcus*, while *Faecalibacterium* was detected at lower abundance. Members of *Prevotella* and *Bacteroides*, exhibiting high sequence similarity constituted the main representatives of Bacteroidota.

Sample T2 showed a broader distribution across the same four major phyla, with Firmicutes (40%) and Bacteroidota (30%) being dominant. Within Bacteroidota, *Bacteroides* and *Prevotella* taxa characterized by strong phylogenetic homology and high 16S rRNA gene similarity were predominant. Firmicutes included *Faecalibacterium*, *Lachnospira*, *Veillonella*, *Agathobacter*, and *Blautia* genera. Actinobacteria were represented by *Brevibacterium*, *Saccharopolyspora*, *Streptomonospora*, and *Nocardiopsis*, while Proteobacteria consisted mainly of members of the Enterobacterales, reported as *Escherichia–Shigella* due to the high sequence similarity.

In sample T3, Proteobacteria (23%) were again dominated by Gammaproteobacteria, with *Pseudoescherichia* emerging as the most abundant genus. Additional Proteobacterial genera included *Klebsiella*, *Acinetobacter*, and *Stenotrophomonas*. Firmicutes (42%) were dominated by *Staphylococcus* and *Streptococcus*, alongside *Paenibacillus*, *Aerococcus*, *Macrococcus*, and *Faecalibacterium*. Actinobacteria were represented mainly by *Brevibacterium* and *Pseudonocardia*, whereas Bacteroidota comprised *Prevotella*, *Phocaeicola*, *Porphyromonas*, and *Bacteroides*.

Sample T4 exhibited the highest genus-level complexity among all samples. Firmicutes (32%), Proteobacteria (29%), Bacteroidota (23%), and Actinobacteria (4%) were all well represented. Within Bacteroidota, *Prevotella* was the dominant genus, with *Porphyromonas*, *Phocaeicola*, and *Bacteroides* also detected, all sharing strong phylogenetic homology within the Bacteroidales order. Proteobacteria showed high taxonomic diversity, including *Pseudoescherichia*, *Aquabacterium*, *Moraxella*, *Caulobacter*, *Acinetobacter*, *Klebsiella*, *Sphingomonas*, and *Methyloversatilis*. Although Fusobacteriota occurred at low overall abundance, *Fusobacterium* exceeded 3% of total reads.

In sample T5, the microbial community was dominated by Firmicutes (42%) and Proteobacteria (28%). *Pseudoescherichia* was the most abundant Proteobacterial genus, followed by *Klebsiella* and *Acinetobacter*. Firmicutes included *Paenibacillus*, *Streptococcus*, *Staphylococcus*, *Aerococcus*, and *Faecalibacterium*. Bacteroidota were represented by *Prevotella*, *Phocaeicola*, and *Porphyromonas*, while Actinobacteria consisted primarily of *Brevibacterium*. Members of Fusobacteriota were detected exclusively as *Fusobacterium*.

In sample T6, Firmicutes (38%) and Proteobacteria (31%) were the most abundant phyla. Gammaproteobacteria included *Stenotrophomonas*, *Enterobacter*, *Serratia*, *Acinetobacter*, and *Pseudomonas* genera. Bacteroidota were represented by *Chryseobacterium*, *Phocaeicola*, *Prevotella*, and *Bacteroides*. Firmicutes comprised *Macrococcus*, *Lactococcus*, *Calidifontibacillus*, *Bacillus*, *Blautia*, *Faecalibacterium*, *Staphylococcus*, *Enterococcus*, and *Priestia*, while Actinobacteria included *Cutibacterium* and *Illumatobacter*.

## DISCUSSION

Previous studies have demonstrated that historical books and manuscripts are complex microbial reservoirs and their microbial assemblages can be characterized using both culture-dependent and culture-independent approaches 16, 33, 35, 36. In the present study, we applied these complementary methodologies to characterize the bacterial communities of six manuscripts from the Süleymaniye Manuscripts Library. While cultivation enabled the recovery of viable bacteria under standard laboratory conditions, Illumina-based sequencing revealed the broader community structure, including taxa that may be non-culturable or no longer viable but represented by residual, fragmented DNA.

The culture-dependent analyses workflow, which incorporated 16S rRNA gene amplification and Sanger sequencing, identified nine species among sixteen isolates. Isolates *Niallia circulans* (formerly *Bacillus circulans*), *Priestia megaterium* (formerly *Bacillus megaterium*), and *Ferdinandcohnia humi* are phylogenetically related to the genus *Bacillus*, a group frequently detected on paper-based substrates and widely recognized for their cellulolytic capabilities 36. *B. circulans* is commonly found in soil, sewage, and food products 37. *Microbacterium foliorum* is predominantly isolated from soil, plants, water, and dairy products 38. Various *Microbacterium* species have been isolated from books 22, 39 and detected by NGS analyses in both parchment and paper manuscripts 14, 34. *Micrococcus luteus* has likewise been reported in paper-based materials through culture-dependent methods 22, 24, and was identified as a dominant species in air samples collected from archival environments 40. The presence of these taxa is consistent with previous studies highlighting the propensity of paper substrates to harbor environmental and handling-associated bacteria with potential roles in biodeterioration.

In metagenomic analyses, high levels of OTU richness were observed at the genus level. In addition, our analysis strategy pooled OTUs below 1% into an “other” category. The NGS data obtained in this study provided important insights into the potential sources of contamination through the characterization of bacterial community profiles. The high richness and diversity of bacterial OTUs may be influenced by multiple factors, including the production and material history of manuscripts dating from the 13th to the 18th centuries, their movement across different geographic regions, and repeated handling by numerous individuals over time. The findings are discussed under different topics in relation to previous studies, and bacterial related biodeterioration assessment are addressed.

### Dominant genera related with biodeterioration process

In sample T1, the genus *Halomonas* was identified as the most abundant taxa. Members of this genus are halophilic bacteria that require high salt concentrations for growth and have previously been linked to the deterioration of parchment and leather materials 31, 41. The dominance of *Halomonas* likely reflects microbial transfer from the leather binding to the paper surface, given the sampling location beneath the leather cover.

*Saccharopolyspora*, identified in samples T1 and T2, have been repeatedly reported as effective agents of collagen degradation and parchment destruction, and have also been identified on paper surfaces in previous studies 13, 30, 41, 42. Saccharopolyspora species inhabit a broad range of ecological niches, including soil, marine sediments, plant-associated environments, and clinical samples 43, reflecting their extensive adaptive capacity and their reported occurrence on cultural heritage materials affected by biodeterioration.

The genus *Nocardiopsis*, identified in sample T2, has previously been associated with the deterioration of parchment manuscripts 28, 44. Although species of *Nocardiopsis* have been isolated from a variety of ecological niches, they are predominantly soil-dwelling microorganisms and are known to produce a range of extracellular enzymes, including amylases, chitinases, cellulases, and β -glucanases 45. In addition, many species within this genus exhibit high tolerance to alkaline and saline conditions, as well as to desiccation 46, 47. Collectively, these adaptive traits may facilitate the persistence of *Nocardiopsis* on historical materials such as parchment and potentially on paper-based substrates.

Notably, sample T4 was the only manuscript in which the genus *Caulobacter* exceeded 1% relative abundance, with *Caulobacter segnis* and *Caulobacter mirabilis* as dominant representatives. Species of this genus have been reported to possess the ability to degrade cellulose and lignin and to grow on cellulose-containing substrates 48. Pinar *et al.* 49 reported that the presence of *Caulobacter* on historical paper materials may constitute a risk for biodeterioration due to their cellulolytic capabilities.

### Identification of genera considered as the core paper microbiome

Bacterial taxa previously reported in the literature as components of the core paper microbiome were identified in the present study. Across the samples, genus-level profiling revealed the presence of *Bacillus*, *Pseudomonas*, *Stenotrophomonas*, and *Acinetobacter*. These genera are widely recognized in the literature as persistent colonizers of historic paper and archival materials and are frequently implicated in biodeterioration processes due to their metabolic versatility and capacity to secrete extracellular enzymes under favorable microclimatic conditions 10, 14, 21, 28, 30, 32, 35, 49.

*Bacillus* species possess pronounced cellulolytic and proteolytic activities, and their metabolic products—including pigments—have been linked to staining phenomena and foxing patterns observed on historical paper documents 22, 23. In addition to *Bacillus*, several phylogenetically related genera belonging to *Bacillus*-group lineages were detected across the samples, including *Paenibacillus* (T2–T3), *Brevibacillus* (T4), *Priestia* (T6), and *Calidifontibacillus* (T6). These taxa are members of stress-tolerant, spore-forming *Firmicutes* that are commonly reported from historic paper materials. Their widespread occurrence supports the notion that *Bacillus*-related lineages constitute an important ecological fraction of paper-associated microbial communities. Notably, *Brevibacillus* has previously been associated with foxing spots on historical paper substrates, further reinforcing the link between this lineage and visible deterioration phenomena 32.

The genus *Pseudomonas*, detected in sample T6, represents another well-documented member of the core paper microbiome. Numerous NGS-based studies have reported *Pseudomonas* species on paper, parchment, and other cellulose-rich heritage materials 21, 32, 33, 49, 50. Experimental studies have demonstrated that *Pseudomonas* spp. are capable of degrading key paper constituents, including starch, casein, and carboxymethylcellulose, highlighting their functional relevance in biodeterioration processes 51. Pangallo *et al.* 52 isolated *Pseudomonas* species from fresco surfaces and the surrounding indoor air, highlighting their ecological versatility and potential role in biodeterioration processes.

*Acinetobacter*, detected in samples T3–T6, is consistently reported in manuscript microbiome studies 21, 49, 50. Species belonging to this genus are ubiquitous in soil and aquatic environments and exhibit strong surface-adherence capabilities, resistance to desiccation, and tolerance to nutrient-poor conditions 53, 54. The occurrence of *Acinetobacter* species on paper-based materials and books can be attributed to these physiological and ecological characteristics of the bacteria.

Members of the genus *Stenotrophomonas* were detected at high relative abundance in sample T6 and at lower abundance in T3. This genus has previously been associated with both parchment manuscripts and historical paper documents 10, 14, 28, 30. Their biodeteriorative potential has been attributed to the secretion of extracellular enzymes such as proteases and chitinases, as well as their strong biofilm-forming capacity mediated by extracellular polysaccharides, which enhance adhesion to solid substrates 49. *Stenotrophomonas rhizophila*, identified at the species level in T6, exhibits endoglycosidase activity that has been shown to contribute directly to cellulose degradation 55. In contrast, sequences assigned to *Stenotrophomonas* in T3 were predominantly affiliated with *S. maltophilia*, a metabolically versatile species commonly isolated from soil, tap and spring water, plants, and indoor environments 56.

Taken together, the detection of metabolically versatile, stress-tolerant, and enzyme-producing bacterial genera across multiple manuscript samples in the present study is consistent with the paper-associated core microbiome reported in the literature. These taxa may colonize paper substrates at various stages of a manuscript’s lifecycle from production and handling to storage and long-term preservation through repeated exposure to air, dust, soil, and water, largely independent of geographic location. Their widespread occurrence and documented biodeteriorative capabilities indicate their relevance for understanding long-term microbial dynamics on historic paper and for developing evidence-based conservation strategies.

### Human contact as dominant contamination source

A major ecological sign from the genus-level community profiles is the representation of taxa frequently associated with the human microbiome, which is consistent with human contact being a contamination source for manuscripts that were historically handled and consulted. Genera such as *Staphylococcus* and *Streptococcus* were widespread across the samples and frequently dominated the bacterial communities, with *Staphylococcus* detected at relative abundances below 1% only in sample T2 and *Streptococcus* below 1% only in sample T1. The genus *Staphylococcus* is part of the human skin microflora and has been associated with human contact in books. 21, 22, 34, 44, 49, 50 Members of the genus *Streptoccocus* are among the bacteria that form the core microbiome of human saliva and the ocular surface 57, 58.

In sample T4, the majority of sequences assigned to the genus *Moraxella* were identified as *Moraxella osloensis*, a saprophytic species commonly found on human skin and mucous membranes and known to be associated with upper respiratory tract infections. *M. osloensis* has previously been identified using next-generation sequencing technologies in Leonardo da Vinci’s drawings and in 19th-century library materials 18, and was also isolated by Jurado *et al.* 29 from letters written by the Pope during the 15th and 16th centuries.

The genera *Brevibacterium* and *Fusobacterium* were detected at relative abundances exceeding 1% in samples T2, T3, and T5, while *Cutibacterium* was identified at >1% in sample T6, *Macrococcus* in samples T3, T4, and T6, and *Veillonella* in sample T2. Members of the genus *Brevibacterium* have previously been isolated from parchment manuscripts in several studies 12, 42 and are also commonly found as constituents of the human skin microbiota. *Cutibacterium* (formerly *Propionibacterium*) is a well-established member of the human skin flora and has been identified on paper surfaces by Pinar *et al.* 49 and Pavlovic *et al.* 15. It has been reported that the propionic and acetic acids produced by *Cutibacterium* species contribute to the characteristic odor often observed in aged books 15.

Members of the genus *Agathobacter*, detected in sample T2, are predominantly associated with the human oral mucosa and various body sites 59. In addition, *Fusobacterium* species identified in samples T3 and T5 are typical inhabitants of the human oral cavity and gastrointestinal tract, further supporting a human-associated origin for these bacterial taxa 60.

Beyond these well-established human microbiome indicators, additional genera commonly encountered in human-associated or indoor environments were detected. These included *Aerococcus* (T3–T5), which is frequently reported in association with human skin and mucosal habitats 60.

### Water-related contamination signs

In addition to contamination signs associated with human handling and environmental exposure, the genus-level community profiles revealed the presence of bacterial taxa indicative of dirty water related contamination. Several of the detected genera are well established members of the human intestinal microbiome and are commonly used as biomarkers of sewage or wastewater impacted environments, suggesting that exposure to contaminated water sources may have contributed to the microbial assemblages observed on the manuscripts.

Our profiles contained signs of dirty water linked contamination or wetting events, indicated by the high relative abundances of gut-associated genera across multiple samples. Genera including *Prevotella* (abundant), *Faecalibacterium* (variable; below 1% only in T3), *Bacteroides* (present in most samples; below 1% in T5), and *Porphyromonas* (T3–T5) were detected at relatively high abundances. Members of the genera *Prevotella* and *Porphyromonas* were identified in historical books by Pavlovic *et al.* 15 using nanopore sequencing technology. Bacteria belonging to the gut microbiota are capable of degrading plant-derived cellulose and converting it into simple sugars through the action of complex cellulolytic enzyme systems. Bacterial taxa exhibiting high cellulase activity may therefore contribute to cellulose degradation in paper and be associated with structural damage.

The genus *Lactococcus* was detected in sample T6, with the majority of sequences affiliated with *Lactococcus lactis* subsp. *cremoris*. This subspecies is primarily associated with the digestive systems of mammals. In addition, various *Lactococcus* species have previously been identified on paper surfaces using next-generation sequencing approaches by Pinar *et al.* 49, further supporting their occurrence on paper-based cultural heritage materials.

In the present study, the high abundance and diversity of gut-associated bacterial taxa detected on paper materials indicate that the manuscripts were extensively exposed to these bacteria and that paper may have served as a suitable growth substrate. These bacterial species are also widely distributed in environmental reservoirs, including water sources, soil, plants, and animal feces (*e.g.*, worms and rodents), suggesting that contamination may have originated from multiple pathways 61.

A further component supporting water-related exposure is the detection of coliform- and Enterobacterales-related genera at low abundance, including *Klebsiella* (T3–T5*), Escherichia–Shigella* (T2), and in T6, *Enterobacter* and *Serratia*, as well as *Enterococcus.*

Culture-dependent analyses provided complementary evidence for water-related contamination. *Niallia circulans* (formerly *Bacillus circulans*) was isolated from samples T1, T2, and T3 using cultivation-based methods. This species has been repeatedly reported from sewage, wastewater, and soil environments influenced by contaminated water, and is recognized for its ability to form highly resistant endospores. Given that the studied manuscripts have been stored in library storage areas since the 1970s, it is plausible that *N. circulans* was introduced to water exposure during earlier periods, such as contact with contaminated tap water, leaks, or damp storage conditions, and subsequently persisted on manuscript surfaces as dormant spores over prolonged periods.

Finally, multiple Proteobacteria consistent with aquatic or water-system associations were detected in the most taxonomically complex sample (T4), including *Aquabacterium*, *Caulobacter, Sphingomonas,* and *Methyloversatilis,* alongside *Moraxella, Acinetobacter*, and *Klebsiella*. In particular, *Aquabacterium* has been previously linked to restoration-related water exposure (*e.g.*, steam-based cleaning practices) 15 and is generally considered unlikely to be a primary biodeterioration driver due to limited reported cellulolytic potential 62; its presence therefore may reflect secondary colonization introduced through water contact rather than active degradation.

### Bacterial footprint providing insights into papermaking materials

The genus *Pseudonocardia* was detected at low relative abundance in sample T3. Members of this genus are known to inhabit diverse ecological niches and are frequently isolated from soil as well as from the roots and leaves of various plant species 63. Most sequences in T3 were assigned to *Pseudonocardia broussonetiae* at the species level. *P. broussonetiae* was originally isolated from the roots of *Broussonetia papyrifera* (paper mulberry), a plant historically associated with papermaking 64. Although DNA degradation and fragmentation occur during papermaking processes—especially those involving heat or chemical treatments—plant DNA can persist on the finished paper. In a DNA-based study comparing different extraction protocols, *B. papyrifera* DNA was successfully isolated from an early 20th-century paper produced from this plant 65. This finding demonstrates that residual plant-derived DNA can survive on a range of plant-based writing materials.

The detection of *P. broussonetiae* in sample T3 suggests a potential association with *B. papyrifera*, raising the possibility that paper mulberry may have contributed to the raw material used in the production of the manuscript’s paper. Paper mulberry has long been utilized in Chinese papermaking traditions, and microscopic analyses have identified its fibers in 10th-century Chinese rag papers 66. It has been proposed that such fibers could derive from clothing used as rag material or from pre-manufactured paper sheets incorporated through the tapa technique. These papers are believed to have been produced by Chinese papermakers in later historical periods. Although Islamic papermaking relied predominantly on rags as the primary raw material, historical records indicate that Chinese paper was transported into Islamic regions during the 15th century for use in manuscript production 67. Furthermore, extensive trade connections between China and the Islamic world via the Silk Road facilitated the movement of papermaking technologies and materials 68. Considering this historical context, it is plausible that paper mulberry fibers—whether in the form of rags, garments, or imported Chinese paper—may have reached Islamic territories and contributed to the paper used in manuscript T3.

### Methodological biases underlying divergence between culture-dependent and culture-independent datasets

In culture-dependent analyses, only those bacteria capable of growing under the specific laboratory conditions provided (*e.g.*, temperature, relative humidity, and culture medium) can be isolated. In the present study, it is likely that only viable and culturable bacteria were recovered, whereas VBNC bacteria did not form detectable colonies on the selected media under standard incubation conditions. Several factors—including the sampling location, the sampling technique, the incubation conditions (such as the predominance of competitively superior species during nutrient broth incubation), and the choice of culture media—can substantially influence the outcome of culture-dependent assays. Consequently, metagenomic sequencing revealed a substantially more diverse and complex bacterial community than that captured through cultivation alone. Although NGS offers high sensitivity and a broad taxonomic resolution, a limitation of this approach is that genera or species present at very low relative abundances may remain undetected or may not be directly associated with the observed deterioration of the artifacts 16, 33.

The divergence observed between culture-dependent and culture-independent datasets in this study primarily reflects well-recognized methodological biases inherent to each approach. In our dataset, this methodological complementarity is illustrated by the frequent recovery of Bacillus-lineage isolates (*e.g.*, *Niallia, Priestia*, and *Bacillus*) alongside the broader NGS-detected assemblage containing gut-associated anaerobes and diverse Proteobacteria. NGS-based culture-independent analyses provide a broader snapshot of community composition, capturing both dominant and rare taxa, including non-cultivable or dormant microorganisms. Accordingly, NGS analyses in the present study revealed high bacterial diversity, from which dominant taxa were highlighted to reflect the most ecologically relevant groups associated with biodeterioration. However, sequencing-based approaches are also subject to biases, such as DNA extraction efficiency, primer specificity, PCR amplification bias, and variation in rRNA gene copy number, which can influence relative abundance estimates.

Notably, several bacterial taxa isolated through cultivation were detected at low relative abundance in the NGS datasets. This apparent discrepancy may indicate that these organisms, although numerically minor within the community, are metabolically active and capable of proliferation under favorable conditions, thus becoming detectable through cultivation. Such taxa may play disproportionately important functional roles in biodeterioration processes despite their low abundance in sequencing data.

By integrating culture-dependent and culture-independent approaches, this study underscores the complementary nature of the two methodologies, enabling both broad community profiling and functional interpretation of bacterial taxa associated with material deterioration. The combined use of these methods therefore provides a more robust framework for understanding microbial involvement in biodeterioration.

## CONCLUSION

In this study, we characterized bacterial communities associated with biodeteriorated regions of six historical manuscripts using complementary culture-dependent and metagenomic approaches. Next-generation sequencing revealed substantially higher bacterial richness and diversity than culture-based methods alone, highlighting the importance of culture-independent analyses for documenting the full microbiome of heritage materials.

Our data show that, although a core set of paper-associated genera—such as Bacillus, Stenotrophomonas, Pseudomonas and Acinetobacter—is recurrent across samples, each manuscript harbors a distinct bacterial community shaped by its age, material composition, handling history, and storage conditions. The high relative abundances of gut-associated and human-related taxa further indicate intense historical human interaction and suggest that contamination pathways often involve water, dust, and vectors such as insects and rodents. These findings reinforce the need to evaluate bacterial biodeterioration on an individual, manuscript-based basis rather than assuming uniform microbial risks across collections.

The combined use of culture-dependent isolation and Illumina-based metagenomic sequencing provided a robust framework for identifying both culturable and non-culturable taxa and for inferring their potential roles in biodeterioration. Importantly, the detection of Pseudonocardia broussonetiae—an endophyte of paper mulberry (*Broussonetia papyrifera*)—suggests that this plant may have contributed to the raw materials used in one of the manuscripts and offers new evidence for the use of paper mulberry in Islamic paper production. This hypothesis should be further tested through advanced fiber analyses and archival studies, including trade records.

Overall, the bacterial “archives” preserved in manuscripts dating from the 13th to the 18th century provide valuable information on past environments, materials, and conservation histories. A better understanding of the ecology, survival strategies, and degradation mechanisms of destructive microorganisms will help refine conservation treatments and improve long-term preservation strategies. In addition, because several identified taxa include opportunistic human pathogens, appropriate health and safety precautions should be taken when handling these artifacts.

## MATERIALS AND METHODS

### Manuscripts

Six manuscripts from two different collections housed in the Süleymaniye Manuscripts Library (Istanbul, Türkiye) were selected for analysis due to the presence of visible biodeterioration. Based on their production dates and visual inspection, all manuscripts appear to have been manufactured using handmade paper. The manuscripts belonging to the Serres Collection and the Mehmet Hilmi–Fethullah Fehmi Collection possess comparable historical and cultural value. The Mehmet Hilmi–Fethullah Fehmi Collection was donated to the Süleymaniye Manuscripts Library in 1970 by the grandchildren of Mehmed Hilmi and Fethullah Fehmi, both of whom served as muftis in Serres. The manuscripts of the Serres Collection were gathered from libraries and madrasahs in and around Serres during the Balkan War. They were initially stored in the Sultanahmet Mosque in Istanbul and were subsequently transferred to the Süleymaniye Manuscripts Library in 1925. Almost all of the examined manuscripts exhibited similar biodeterioration symptoms, including discoloration, detachment of outer layers, tearing, and loss of marginal paper sections. Since 2012, these manuscripts have been preserved in the controlled storage areas of the Süleymaniye Manuscripts Library, where environmental conditions are maintained at 18–20 ${}^{\circ }$ C and 45–55% relative humidity using compact shelving systems. (Detailed information regarding the investigated manuscripts is presented in Table 3.)

**Table 3:** Textual information of the analyzed manuscripts and description of biodeteriorated areas .

**Collection Name and Number**	**Sampling Code**	**Textual Information and Date**	**Deteriorated parts**
**M. Hilmi F. Fehmi Collection, No: 54**	T1	*The Book of Fatwas* by Şeyhülislam Çatalcali Ali Efendi. No exact date available; presumed to have been written between the 17th–18th centuries	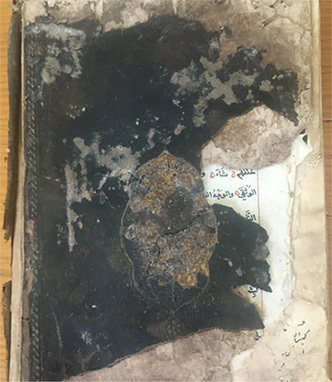
			Downy deterioration; tears; detachment and missing paper sections; pink stain formation on both leather and paper; losses on leather.

**M. Hilmi F. Fehmi Collection, No: 231**	T2	A manuscript on Arabic literature. Written in **1613**.	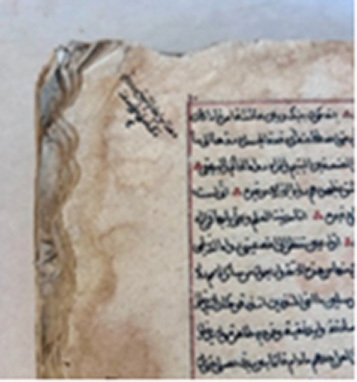
			Downy deterioration or fiber disruption along the lower edge; missing paper sections and tears; signs of rodent damage.

**M. Hilmi F. Fehmi Collection, No: 17**	T3	A reference book among the fatwa manuscripts. Written in **1422**.	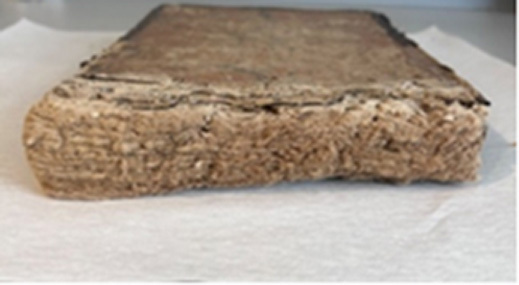
			Large losses of paper at the upper edge; downy deterioration; stains on paper.

**M. Hilmi F. Fehmi Collection, No: 71**	T4	A reference book among the fatwa manuscripts. Written in **1502**.	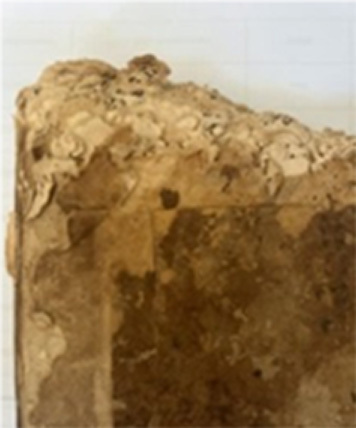
			Large losses parts of paper at the upper edge; downy deterioration or fiber disruption; missing sections within the text block.

**M. Hilmi F. Fehmi Colleciton, No: 90**	T5	A significant fatwa manuscript. Written in **1490**.	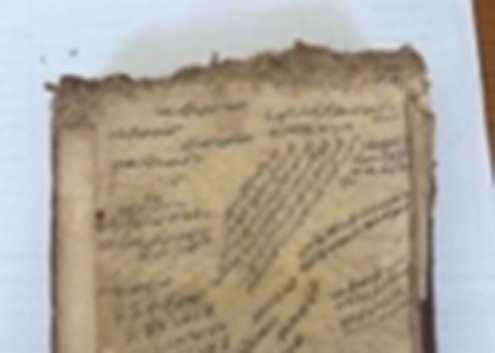
			Large losses at the upper edge; downy deterioration or fiber disruption; stains on paper,

**Serezli Collection, No: 3647**	T6	A manuscript on Arabic literature. Written in **1237**.	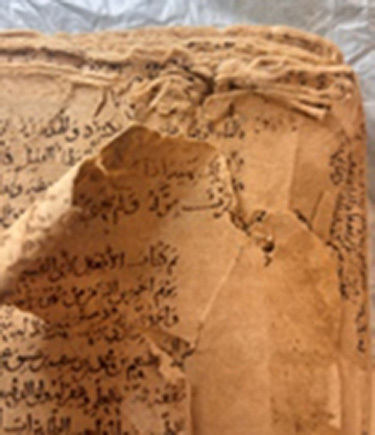
			Downy deterioration at the upper edge; missing paper sections and tears.

This table summarizes the collection names and numbers, sampling codes (T1–T6), textual information including manuscript content and estimated dates, and the main types of biodeterioration observed in each manuscript. Documented deterioration features include downy deterioration or fiber disruption, paper losses and tears, missing sections, staining, rodent damage, and deterioration of leather components. The descriptions are based on in situ visual assessment conducted prior to sampling and conservation procedures.

### Sampling

**Figure 3:**
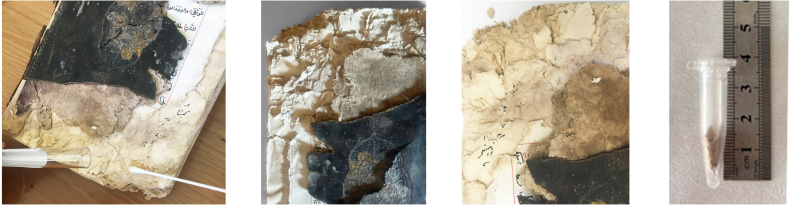
Sampling procedures and material preparation for culture-dependent and metagenomic analyses of historic manuscripts. **(A)** Sampling of manuscript surfaces using sterile swabs for culture-dependent microbiological analyses. **(B)** Paper fragments collected from deteriorated areas of the manuscript and prepared for culture-independent metagenomic analyses. Sampling was performed under controlled conditions to minimize damage to the original material and to prevent external contamination.

Samples for the culture-dependent analysis were collected from biodeteriorated areas using sterile swabs (Cultiplast, LOT: Z1080A). For the metagenomic (culture-independent) analysis, sampling was performed using paper fragments that had spontaneously detached as a result of advanced biodeterioration and were unsuitable for restoration. These paper fragments were obtained exclusively from unwritten marginal areas under sterile conditions to prevent contamination and preserve the integrity of the textual content. All fragments were immediately transferred into 1.5 ml sterile microcentrifuge tubes for subsequent molecular analysis. For each manuscript, samples used for culture-dependent and culture-independent analyses were collected from the same deteriorated regions. This approach ensured that both methodologies targeted comparable micro-environments within each manuscript. (Figure 3)

### Culture dependent analysis

Sterile swabs were inoculated into 5 mL tubes containing Nutrient Broth (Merck, 105443). The tubes were incubated in a shaking incubator at 150 RPM and 26 ± 1 ${}^{\circ }$ C for 24 hours. Following incubation, the NB cultures were streaked onto Tryptic Soy Agar (TSA; Merck, 105458) using the spread-plate technique. Plates were incubated at 26 ± 1 ${}^{\circ }$ C for 7 days. Pure bacterial colonies were obtained by repeated subculturing of individual colonies from mixed cultures.

For the detection and isolation of *Escherichia coli* and other coliform bacteria, sampling swabs were also directly streaked onto Chromogenic ECC Agar (Laborlar, LB.709; LOT: 1003). These plates were incubated at 36 ${}^{\circ }$ C for 48 hours. All sampling and inoculation procedures were performed in triplicate.

Colony morphology of the isolated bacteria was recorded, and Gram staining was performed for preliminary morphologic characterization. For molecular identification, the 16S rRNA gene was selected as the target for amplification and sequencing. Genomic DNA from pure cultures was extracted using the ZYMO Research Quick-DNA Fungal/Bacterial Miniprep Kit (Catalog No. D6005), following the manufacturer’s standard protocol.

Universal primers 27F (5’-AGAGTTTGATCMTGGCTCAG-3’) and 1492R (5’-TACGGYTACCTTGTTACGACTT-3’) were used to amplify the 16S rRNA gene of each isolate. The PCR reaction mixture (final volume: 35 μ L) consisted of 12.5 μ L 1 × FIREPol® DNA Polymerase Master Mix (Solis BioDyne), 5 μ L forward primer, 5 μ L reverse primer, 3 μ L template DNA, and nuclease-free water. PCR amplification was conducted on a Kyratec thermocycler using the following cycling conditions: initial denaturation at 95 ${}^{\circ }$ C for 5 min, 30 cycles of Denaturation at 94 ${}^{\circ }$ C for 45 s, Annealing at 57 ${}^{\circ }$ C for 45 s, Extension at 72 ${}^{\circ }$ C for 60 s, and a Final elongation step 72 ${}^{\circ }$ C for 5 min. PCR products were visualized on 1.5% agarose gel electrophoresis stained with ethidium bromide. Successful amplicons were sequenced using Sanger sequencing by a commercial service provider (BM Laboratory Systems, Ankara, Türkiye). Raw sequence data were aligned, and chimeric reads were removed using BioEdit. The obtained sequences were compared against reference sequences in the NCBI GenBank database using the BLAST algorithm to determine taxonomic identity.

### Metagenomic analyses

High-throughput sequencing using the Illumina MiSeq platform was conducted to characterize the microbial communities associated with the six manuscript samples. DNA was extracted from paper fragments using the GeneMATRIX Tissue & Bacterial DNA Purification Kit (EurX, Cat. No. 3550), following the manufacturer’s standard protocol. DNA concentrations were quantified using a VICTOR3 Fluorometer with PicoGreen dye. The V3–V4 hypervariable regions of the bacterial 16S rRNA gene were amplified using V3 forward primer (5’TCGTCGGCAGCGTCAGATGTGTATAAGAGACAGCCTACGGGNGGCWGCAG-3’) and V4 reverse primer (5’GTCTCGTGGGCTCGGAGATGTGTATAAGAGACAGGACTACHVGGGTATCTAATCC-3’) primer sets. Total volume of 25 μ L reaction mixture of first PCR contained 12.5 μ L 2X KAPA HiFi HotStart Ready Mix (Kapa Biosystems), 5 μ L reverse primer, 5 μ L forward primer, 2.5 μ L of the extracted DNA. Thermal cycling condition was as follows; initial denaturation at 95 ${}^{\circ }$ C for 3 min, 25 thermal cycles of denaturation at 95 ${}^{\circ }$ C for 30 s, annealing at 55 ${}^{\circ }$ C for 30 s, extension at 72 ${}^{\circ }$ C for 30 s and a final elongation at 72 ${}^{\circ }$ C for 5 min. PCR Amplicons were purified using AMPure XP beads (Beckman Coulter) according to the manufacturer’s protocol. Purified products were visualized by electrophoresis on 1.2% agarose gels.

Library preparation was performed using purified amplicons following the standard Illumina MiSeq library preparation workflow. Indexing PCR was performed using “Nextera XT index kit”. First PCR products were used as template DNA for indexing PCR and 2 index barcode regions were annealed. Total volume of 50 μ L reaction mixture of indexing PCR contained 25 μ L 2X KAPA HiFi HotStart Ready Mix, 5 μ L Nextera XT index primer 1(N7xx), 5 μ L Nextera XT index primer 2 (S5xx), 5 μ L template DNA and 10 μ L sterile water. Index PCR protocol was as follows: initial denaturation at 95 ${}^{\circ }$ C for 3 min, 8 thermal cycles of denaturation at 95 ${}^{\circ }$ C for 30 s, annealing at 55 ${}^{\circ }$ C for 30 s, extension at 72 ${}^{\circ }$ C for 30 s and a final elongation at 72 ${}^{\circ }$ C for 5 min. The products of index PCR were purified using 56 μ L AMPure XP beads, ensuring the removal of residual primers and primer dimers.

Sequencing was performed using Sequencing-by-Synthesis (SBS) technology on the Illumina MiSeq platform by a commercial service provider (BM Laboratory Systems, Ankara, Türkiye). Raw FASTQ reads were submitted in the Sequence Read Archive (SRA) section of the NCBI database (SRA accession number: SAMN42359052; Bioproject no: PRJNA1132802).

Raw Illumina FASTQ data were converted to FASTA format and an initial quality assessment was performed using FastQC to evaluate read counts, GC content, quality scores, and read length distributions. Subsequent bioinformatic analyses, including quality filtering of raw reads, removal of chimeric sequences, merging of paired-end reads, and filtering of barcodes and primers, were conducted using QIIME 2. Reads with Phred quality scores below 20 were excluded from further analyses. High-quality sequences were clustered into OTUs based on 97% sequence similarity. Taxonomic assignment of OTUs was carried out using the BLAST algorithm against the NCBI GenBank database. Species-level identification was applied only to OTUs showing 100% sequence identity with reference sequences in curated databases; OTUs not meeting this criterion were conservatively assigned at the genus level. The bacterial community structure and composition were analyzed using QIIME on the normalized OTU dataset. Krona and Bar plots were generated to visualize community composition and the relative abundance of identified taxa.

## CONFLICT OF INTEREST

The authors declare that they have no conflict of interest.

## ABBREVIATIONS

DGGE –

Denaturing Gradient Gel Electrophoresis –

NGS – next-generation sequencing

OTUs – operational taxonomic units

VBNC – viable-but-non-culturable
